# Tough Bioplastics from Babassu Oil-Based Acrylic Monomer, Hemicellulose Xylan, and Carnauba Wax

**DOI:** 10.3390/ijms24076103

**Published:** 2023-03-23

**Authors:** Yehor Polunin, Vasylyna Kirianchuk, Najah Mhesn, Liying Wei, Sergiy Minko, Igor Luzinov, Andriy Voronov

**Affiliations:** 1 Department of Coatings and Polymeric Materials, North Dakota State University, Fargo, ND 58105, USA; 2 Department of Materials Science and Engineering, Clemson University, Clemson, SC 29634, USA; 3 Nanostructured Materials Laboratory, University of Georgia, Athens, GA 30602, USA

**Keywords:** bioplastic, xylan, babassu oil, carnauba wax, food packaging, moisture barrier, polyethylene replacement

## Abstract

We describe here the fabrication, characterization, and properties of tough bioplastics made of a babassu oil-based acrylic polymer (PBBM), hemicellulose xylan grafted with PBBM chains, and carnauba wax (CW). The plastic was primarily designed to obtain bioderived materials that can replace low-density polyethylene (LDPE) in certain food packaging applications. To obtain plastic, the radical polymerization of an original babassu oil-based acrylic monomer (BBM) in the presence of xylan macromolecules modified with maleic anhydride (X-MA) was conducted. The polymerization resulted in a material (PBBM-X) mostly consisting of highly branched PBBM/X-MA macromolecules. PBBM-X has a glass transition of 42 °C, a storage modulus of 130 MPa (at 25 °C, RT), and a Young’s modulus of 30 MPa at RT. To increase the moduli, we blended PBBM-X with carnauba wax, a natural material with a high modulus and a melting temperature of ~80 °C. It was found that PBBM-X is compatible with the wax, as evidenced by the alternation of the material’s thermal transitions and the co-crystallization of BBM side alkyl fragments with CW. As a result, the PBBM-X/CW blend containing 40% of the wax had a storage modulus of 475 MPa (RT) and a Young’s modulus of 248 MPa (RT), which is close to that of LDPE. As polyethylene, the PBBM-X and PBBM-X/CW bioplastics have the typical stress-strain behavior demonstrated by ductile (tough) plastics. However, the bioplastic’s yield strength and elongation-at-yield are considerably lower than those of LDPE. We evaluated the moisture barrier properties of the PBBM-X/(40%)CW material and found that the bioplastic’s water vapor permeability (WVP) is quite close to that of LDPE. Our bioderived material demonstrates a WVP that is comparable to polyethylene terephthalate and lower than the WVP of nylon and polystyrene. Taking into account the obtained results, the fabricated materials can be considered as polyethylene alternatives to provide sustainability in plastics production in the packaging areas where LDPE currently dominates.

## 1. Introduction

Here we describe bioplastics consisting of a babassu oil-based acrylic polymer (PBBM), hemicellulose xylan grafted with PBBM chains, and carnauba wax (CW). The plastic was primarily designed to obtain bioderived materials that can replace polyethylene (PE) in certain food packaging applications (such as moisture barrier coating). Having quite unique thermo-mechanical properties and being low-cost, PE belongs to the family of olefinic polymers, which are widely used in a number of practical applications [1]. Today, PE is the largest among those plastics in terms of manufactured volumes [2]. The polymer’s hydrocarbon (alkane) structure makes this material hydrophobic and chemically inert (no functional groups in the macromolecular backbone) [3]. To this end, PE does not degrade easily, and its recyclability is limited. Only about 5.3% of low-density polyethylene (LDPE) and 10.3% of high-density polyethylene (HDPE) are recycled [4]. Therefore, the distinctive properties of PE materials on the one hand and growing environmental challenges on the other triggered an interest in and the design of “PE-like” materials based on non-petroleum-based (natural) renewable resources that can be inherently biodegradable or compostable [5,6,7,8,9]. The materials can be obtained from renewable biological resources, for instance, from biomass (including plants), thus leaving no toxic residues upon composting (biodegradation) [10,11]. To display performance comparable to that of polyolefins, biobased polymers must demonstrate certain key physico-chemical characteristics, such as glass transition and melting temperature, thermo-mechanical properties, chemical/moisture resistance, and processability.

To this end, plant/vegetable oils have become increasingly attractive for the formulation of biobased polymers due to oils’ chemical versatility and, often, their abundance and low cost [12,13,14]. We previously reported a one-step method that converts fatty acid esters of plant/vegetable oils into biobased acrylic monomers for free radical polymerization [15,16]. The synthesized plant oil-based monomers (POBMs) offer a unique combination of varying unsaturated and saturated fatty acid fragments, which allows post-polymerization cross-linking of the resulting polymers and simultaneously facilitates the formation of crystalline domains. The combination provides the ability to tailor the thermo-mechanical properties and performance of POBM-based polymeric materials. However, thermoplastic (uncross-linked) materials made from POBMs have a modulus that is significantly lower than that of PE and other semicrystalline polyolefins [17,18]. Thus, in this work, we combined hemicellulose-derived rigid xylan macromolecules with PBBM (a polymer made from POBM synthesized from babassu plant oil) using free radical polymerization to obtain a PBBM/PBBM-grafted xylan mixture (PBBM-X).

Babassu is one of the most important palm species in Brazil [19,20]. Babassu oil extraction has an annual output of about 57,000 tons but can be straightforwardly increased due to the high productivity of palm trees. The oil is composed of primarily saturated lauric (C12:0), myristic (C14:0), and palmitic (C16) acids, as well as monounsaturated oleic acid (C18:1) [19]. The side-saturated aliphatic fragments in PBBM and, thus, PBBM-X are expected to be able to form crystalline morphological domains and, thus, have better mechanical and barrier properties. Indeed, it is shown that long-side chained vinyl homopolymers having saturated side fragments over ten carbon atoms can form finely ordered microcrystalline structures, with the type of crystals determined by chain length [21,22].

Hemicellulose xylan is a polymer composed of five-carbon sugars and is the second most abundant polysaccharide in plant biomass after cellulose [23,24,25,26]. It constitutes 25–35% of the cell walls of most lignocellulosic feedstocks. Despite their abundance, brittle hemicelluloses have yet to be widely utilized to produce value-added products such as plastics. We have foreseen that, owing to the optimized composition of the mixtures containing polar and rigid xylan backbones and hydrophobic flexible plant oil-based side fragments, thermoplastic materials with mechanical properties resembling PEs can be approached. Indeed, our results show that PBBM-X bioplastic is a processable, film-forming, tough, and elastic material with a relatively high modulus. To further adjust the properties of the bioplastic, we blended the PBBM-X with carnauba wax, a semicrystalline solid material having a relatively high modulus [27,28,29]. CW has already been considered for employment in food preservation applications [30,31]. The resulting blended material demonstrated storage and Young’s moduli close to those typically measured for LDPE. We also compared the moisture barrier properties of food packaging paper covered with polyethylene and the bioplastic developed here. It was found that the moisture barrier properties of the paper covered with the bioplastic were approaching those of the PE-coated paper. Therefore, seeing the obtained results, the fabricated bioderived materials can be considered as polyethylene alternatives to provide plastics production sustainability in various areas where polyethylene currently dominates.

## 2. Results and Discussion

### 2.1. Synthesis of PBBM-X

**Figure 1:**
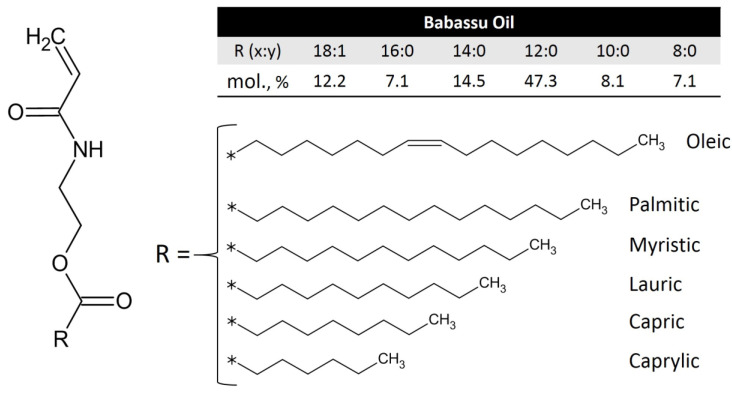
Chemical structure of babassu oil-based monomer (BBM) synthesized in this study. R (x:y) is the structure of the fatty acids (x is the number of carbon atoms in the fatty acid chain, and y is the number of double bonds in the fatty acid). * bond connecting the moiety to the monomer.

It is necessary to note that BBM (Figure 1) is an original monomer synthesized by us, which (to the best of our knowledge) was not previously reported in the scientific literature. In general, we followed a synthetic procedure described in our preceding publications [15,16,32]. Thus, prior to the PBBM-X formation, we studied in detail the structure and homopolymerization of BBM (Supplementary material: Sections S1 and S2). First, we confirmed BBM’s targeted chemical structure using FTIR and ^1^H NMR (Supplementary material: Figure S1). Figure 1 shows the distribution of different-length hydrocarbon side fragments in BBM as determined by mass spectroscopy (Supplementary material: Figure S2). One can see that the monomer has a typical distribution of hydrocarbon fragments for babassu oil. Next, the reactivity of the monomer in free radical polymerization was investigated (Supplementary material: Section S2, Figures S4 and S5). In essence, it is shown that BBM is quite reactive and can be used to synthesize macromolecules.

**Figure 2:**
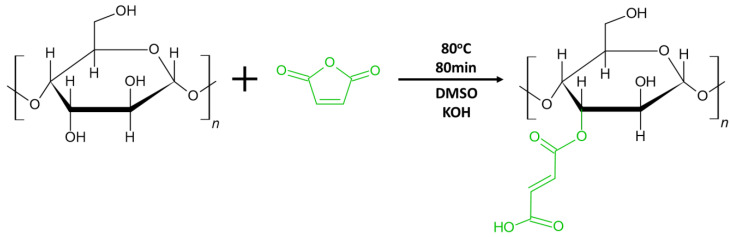
Chemical schematic for maleinization of beechwood hemicellulose xylan.

The PBBM-X was synthesized by radical polymerization, where BBM was polymerized in the presence of xylan chains modified with maleic anhydride (X-MA) using methodology described elsewhere [33]. Thus, before the polymerization, xylan was reacted with maleic anhydride using an esterification reaction (Figure 2). During the maleinization of xylan, an ester bond is formed when maleic anhydride reacts with xylan hydroxyl groups. At the same time, the vinyl group of maleic anhydride is retained to become a reactive site for the attachment of the BBM grafted chains. The ^1^H NMR spectroscopy confirms a successful maleinization (Supplementary material: Section S4, Figure S6). In this work, we used X-MA with a degree of maleinization of 0.3, which determines the number of maleic anhydride moieties per anhydroglucose unit of hemicellulose (Supplementary material: Section S4, Figure S7).

**Figure 3:**
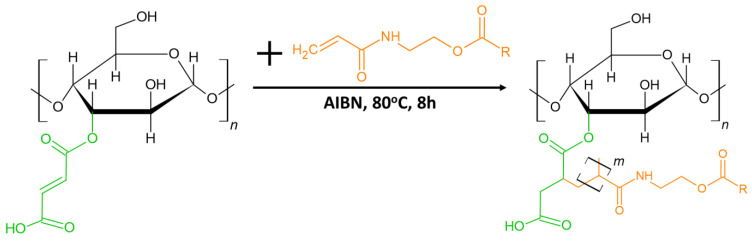
Synthetic scheme of BBM-Xylan grafted copolymerization.

In our polymerization procedure, we added the modified xylan to the BBM bulk containing the polymerization initiator. X-MA is not soluble in BBM. Therefore, initially, the system was highly heterogeneous. As the polymerization proceeded, the polymerizing medium appeared to be more homogeneous; however, some X-MA aggregates were still visible. After the purification described below (Experimental Section), we obtained a homogeneous PBBM-X solution in toluene containing BBM homopolymer and xylan grafted with BBM chains via double bonds of maleic anhydride (Figure 3). We determined gravimetrically from the amount of X-MA aggregates not introduced into PBBM-X that the polymer material contained ~5 wt% of X-MA.

**Figure 4:**
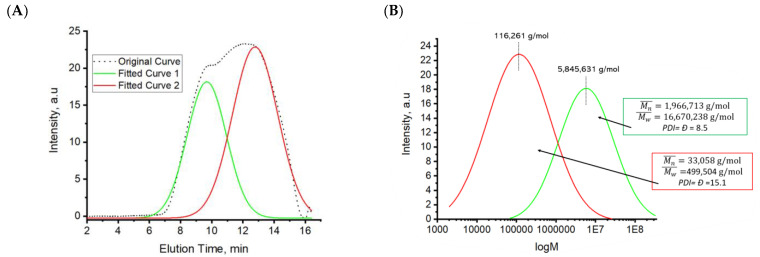
GPC analysis of PBBM-X: (**A**) The original GPC trace deconvoluted into two fractions, and (**B**) the intensity versus molecular weight for the fractions.

We conducted GPC measurements of the polymer’s molecular weight against polystyrene standards (Figure 4). It is evident that there are two major fractions of macromolecules present in the material: (1) the higher molecular weight fraction (MWF) with Mn ≈ 2 M g/mol and a polydispersity (PDI) or dispersity index (*Đ*) of 8.5, and (2) the lower MWF with Mn ≈ 33,000 g/mol and *Đ* ≈ 15.1. The higher and lower MWFs constitute 41% and 59% of the material, respectively. When pure PBBM is synthesized at the same polymerization conditions (in the absence of X-MA), the macromolecules have Mn ≈ 97,000 g/mol and *Đ* ≈ 13. Based on the preliminary study of the homopolymerization of BBM in solution (Supplementary material: Section S2), the molecular weight of the homopolymer in bulk has to be significantly lower than the one found here. The increase in the molecular weight and Đ indicates that at the very high initiation rates used to obtain PBBM and PBBM-X in this work, chain transfer to the polymer chains occurs, yielding branched PBBM macromolecules.

Xylan has a molecular weight between 8000 and 10,000 g/mol. Therefore, we can conclude that PBBM-X consists of highly branched PBBM/X-MA macromolecules, where multiple xylan chains are decorated and connected together with PBBM chains. PBBM homopolymer branched chains are also present in the material and are a major part of the lower molecular weight fraction.

The most prevalent in the low and high MWF are chains with MWs of about 161,000 g/mol and 5.9 M g/mol, respectively. It is possible to estimate the radius of gyration, Rg in bulk, for these macromolecules from the Rg of polystyrene chains used for the GPC calibration [34,35]. The Rg is equal to about 60 nm for the high MWF and 10 nm for the lower MWF. Therefore, one can see that there is a significant difference in the geometrical size of the macromolecules constituting the fractions. We suggest that the lower MWF forms at the first stage of the polymerization. The larger macromolecules are formed later as the smaller ones are connected by newly formed PBBM chains.

### 2.2. Thermal Transitions of Materials

Figure S3 (Supplementary material) shows DSC heating/cooling traces for babassu oil and BBM. Pure babassu oil shows typical behavior for vegetable oils where more than one morphological crystalline form is observed [20,36]. Specifically, three different forms are found, having melting temperatures (Tm) of approximately 8, 13, and 22 °C and crystallization temperatures (Tc) of ~−50, −8, and 5 °C. We did not observe a glass-transition temperature (Tg) for the oil; therefore, the material has a high degree of crystallization. When the monomer is made of babassu oil, the obtained BBM is found to be semicrystalline with a Tg ≈ −10 °C. The melting temperatures for the morphological forms are about 11, 20, and 37 °C, while the crystallization occurs between 10 and −4 °C. The major peak, Tc ≈ 7 °C, has two additional smaller crystallization peaks located between 7 and 10 °C. It is noted that the melting temperatures are significantly higher for the monomer in comparison to the pure oil. We associate this phenomenon with the presence of polar amide and carbonyl groups in BBM capable of forming hydrogen bonds [37,38].

**Figure 5:**
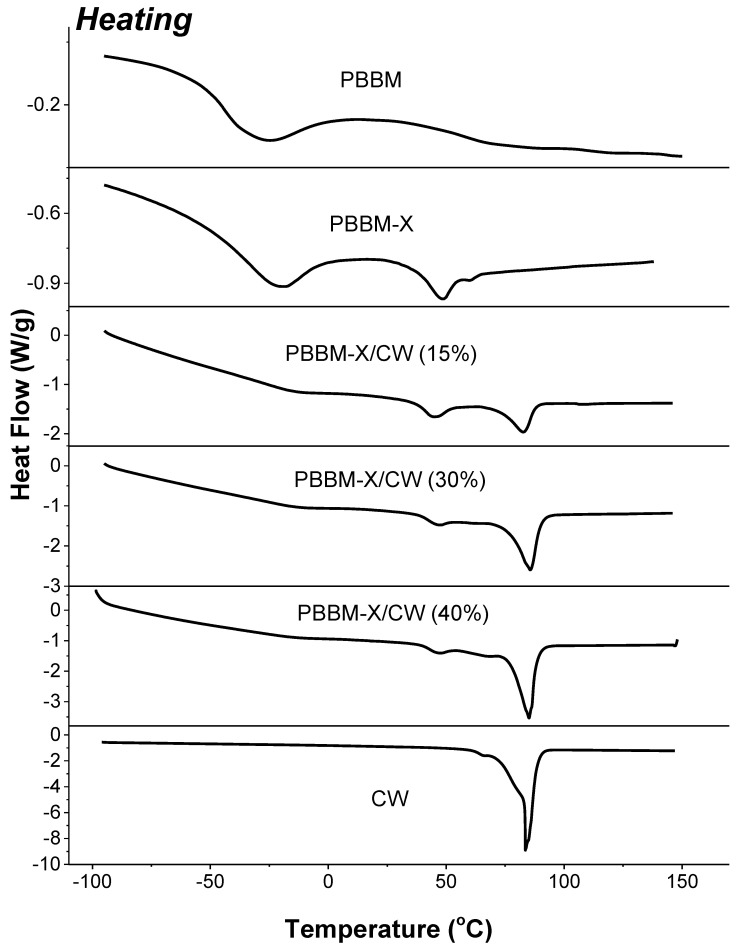
DCS heating traces for the bioplastics.

**Figure 6:**
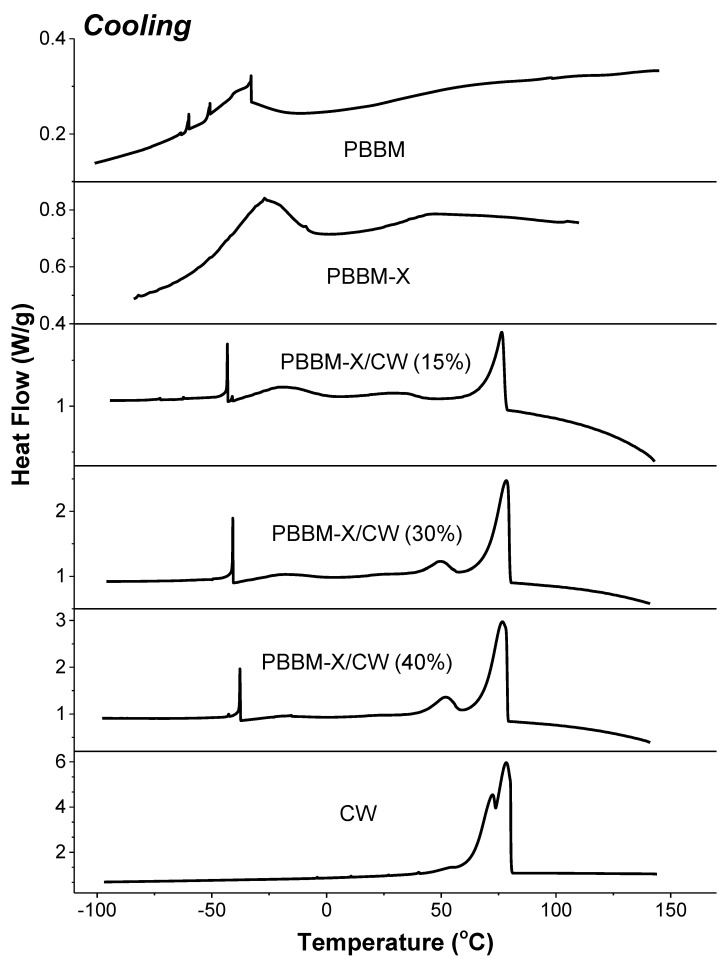
DCS cooling traces for the bioplastics.

Figure 5 and Figure 6 show DCS data for PBBM, the homopolymer made of BBM. It is obvious that the thermal behavior of the obtained macromolecules is drastically different from that of the monomer (Supplementary material: Figure S3). First, the semicrystalline polymer has a wide and blunt melting peak with Tm ≈ −27 °C, about 50 degrees lower than the Tm of BBM. The crystallinity is related to the crystallization of the monomeric unit’s side groups since the melting is well below Tg (observed at 56 °C) and thus cannot be associated with the whole atactic macromolecule. This crystallization of the alkyl side groups is well documented for (meth)acrylates [21,22]. On the other hand, the polymers with relatively long side fragments are shown to demonstrate two distinct glass-transition temperatures: a lower bound Tg1 corresponding to that of the side chain and an upper bound Tg2 corresponding to that of the copolymer main chain [39,40,41,42]. In our case, it appears that Tg1 is relatively close to the Tm of the side groups, and it is difficult to deconvolute the two transitions precisely. However, it is evident that the onset of the side groups Tg is about −78 °C.

When 5 wt% of xylan grafted with BBM is incorporated into PBBM, the thermal transitions resemble the ones observed for PBBM (Figure 5). However, the PBBM-X transitions appeared to be somewhat shifted. The onset Tg for the side groups and the group’s Tm increased to ~ −60 °C and −20 °C, respectively. The main chain Tg, however, decreased to about 40 °C. The obtained result indicated that the presence of about 40 wt% of xylan grafted with BBM macromolecules decreased the mobility of the alkyl side fragments but increased the mobility of the chains constituting the PBBM-X material. We also note that the glass transition for the main chain demonstrates the presence of secondary relaxations [37], which (as evident from the DSC traces) disappear on the controlled cooling.

### 2.3. Thermo-Mechanical Properties of the Materials

**Figure 7:**
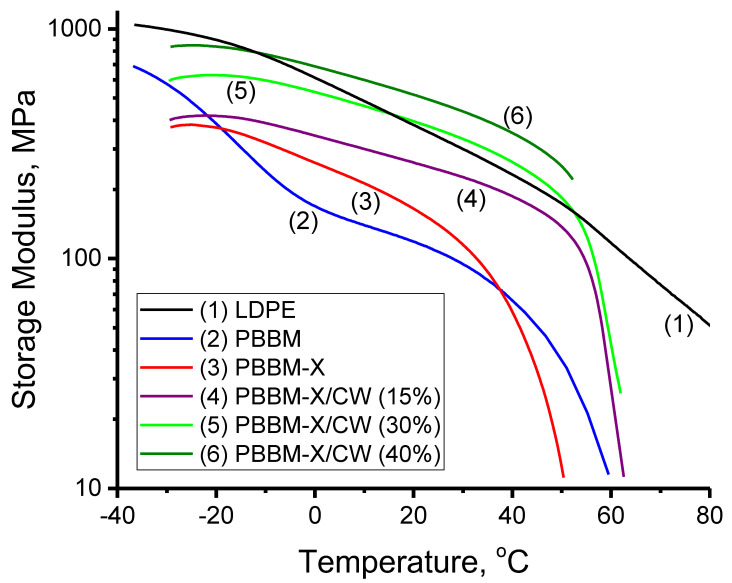
Storage modulus versus temperature for the bioplastics.

DMA was employed to study how the storage moduli of the PBBM and PBBM-X change as a function of temperature (Figure 7). From the DMA data, it is clear that the storage modulus of PBBM is significantly higher at temperatures below the Tg and Tm of the side groups of the monomer. As the side fragments of PBBM undergo the thermal transitions, the modulus sharply decreases between approximately −40 °C and −10 °C and reaches the value of ~108 MPa at room temperature (RT = 25 °C). For PBBM-X, the modulus drop is less pronounced and occurs at higher temperatures. The modulus decreases significantly at about −20 °C and is about 140 MPa at RT. The observed behavior is in accord with our DSC results and confirms that the presence of xylan rigid chains grafted with BBM in the bioplastic decreases the mobility of the side chains. It is also necessary to highlight that the RT storage modulus for PBBM-X is considerably higher (~30%) than that of PBBM. Therefore, introducing less-flexible xylan macromolecules into the BBM-based material improves its mechanical behavior. As the temperature increases further above RT, the storage modulus falls abruptly to approximately 52 °C and 43 °C for PBBM and PBBM-X, respectively. These temperatures are close to the glass-transition temperatures of the polymers determined in the DSC measurements. It is necessary to highlight that a rubbery plateau is not observed for both PBBM and PBBM-X, indicating a low level of entanglements for the polymers obtained [37]. In addition to branching, the low level of entanglements in PBBM is related to the specific structure of the polymer, where long side chains are anchored to the backbone at high densities. Thus, the macromolecules resemble the structure of molecular bottlebrushes, demonstrating a decreased level of entanglements [43,44,45]. PBBM-X has a higher fraction of highly branched polymer chains, which also suppresses the ability of the macromolecules to form entanglements [46,47,48,49].

We also measured the dependence of storage modulus on temperature for the industrial sample of LDPE film at the same experimental conditions as for the bioplastics (Figure 7). The LDPE generally has a somewhat different behavior than the bioplastics studied here, where a more monotonous modulus decrease with the temperature increase is found. The value of the LDPE storage modulus at 25 °C is about 340 MPa, which is close to the typical values reported for the polymer in the scientific literature [50,51,52]. Thus, to modify the thermo-mechanical behavior of the bioplastics to closely follow the behavior of LDPE, we added the higher modulus carnauba wax to the PBBM-X material, a natural material possessing a storage modulus of ~1 GPA at RT with a melting temperature of ~80 °C [27,28,29]. The blends contained 15, 30, and 40 wt% of the wax. It was foreseen that CW could be compatible or miscible with PBBM-X because of the presence of saturated hydrocarbon fragments in both materials. We also envisioned that the side fragments of BBM could co-crystallize with CW, further improving the mechanical and barrier properties of the bioplastic.

### 2.4. Thermal Transitions of PBBM-X/CW Blends

Figure 5 and Figure 6 display DSC curves for PBBM-X/CW blends as well as the ones for the pure wax. According to heating/cooling traces, the wax is a highly crystalline material with three different morphological crystal forms with Tm of about 64, 78, and 85 °C and Tc of ~52, 71, and 77 °C. The material is primarily composed of crystals with higher Tm and Tc, with only a very small fraction of the crystals melting at 64 °C. When the carnauba wax is added to PBBM-X, we still observe the melting and crystallization of the material, but there are considerable variations in the Tm and Tc peak positions. The changes are most accurately detectable in the mixture containing 40% CW, but they are present in all blends studied. First, we observe that there is a pure CW crystalline phase in the bioplastic, with Tm and Tc of ~85 and ~78 °C, the same as for the 100% wax bulk. However, we do not observe the crystals with Tm of 78 °C and Tc of 71 °C in the PBBM-X/CW blends. Instead, a crystallization peak of about 50–53 °C is found. Those crystals are melting at ~62–64 °C.

We associate the changes with the co-crystallization of BBM side groups with CW. Indeed, in the blended bioplastics, the crystallization of the BMM side groups is depressed. It is evident from the significant decrease in the intensity of the crystallization and melting transitions observed for pure PBBM-X located at −25 °C (Tc) and −20 °C (Tm) (Figure 5 and Figure 6). In addition, the glass transition for the monomer alkyl side fragments found for BBM, PBBM, and PBBM-X is not detected for PBBM-X/CW (Figure 5 and Figure 6). We also note an additional sharp (small area) crystallization peak for PBBM-X/CW at approximately −40 °C, which is not present in PBBM-X. From the decrease in the high-temperature melting transition area of CW, we estimated that about 20% of the wax molecules are involved in the co-crystallization. It is necessary to point out that the Tg of PBBM main chains (~42 °C) does not change when PBBM-X is blended with carnauba wax.

### 2.5. Thermo-Mechanical Properties of PBBM-X/CW Blends

**Figure 8:**
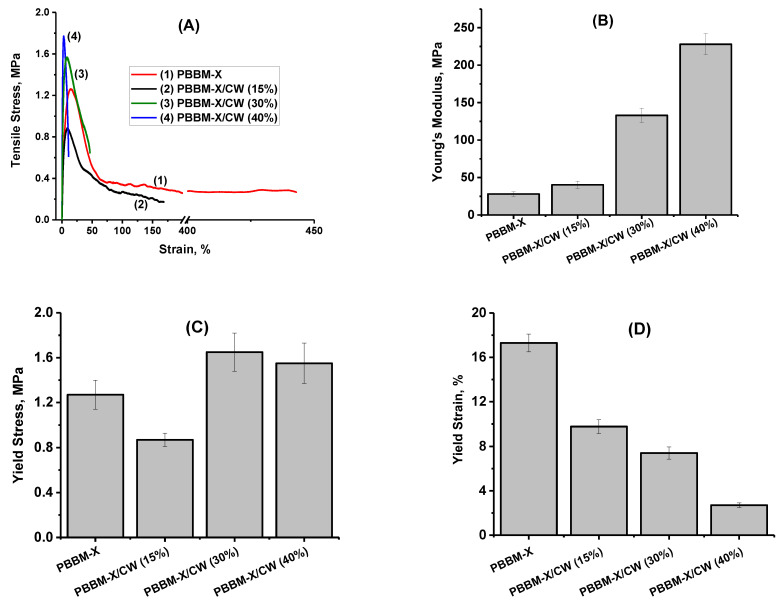
Typical stress-strain curves (**A**), Young’s modulus (**B**), yield stress (**C**), and yield strain (**D**) for the bioplastics.

The results described above demonstrate a significant interaction between the carnauba wax and PBBM-X in the blended materials. Therefore, we have foreseen a considerable influence of CW addition on the thermo-mechanical behavior of bioplastics. Figure 7 shows DMA measurements of the storage modulus for PBBM-X/CW blends of different compositions versus temperature. One can see that CW integration into the bioplastic noticeably increases the value of modulus over the whole temperature range, where the higher modulus is found for the materials with the higher wax content. The storage modulus decreased significantly at about −10 °C and fell sharply at ~52 °C. We note that the CW addition shifts these major transitions to the higher temperatures of −20 °C and 42 °C determined for PBBM-X. The RT modulus for the PBBM-X/CW bioplastics is 245, 360, and 475 MPa for the materials with 15, 30, and 40% addition of the wax, respectively. One can see that the storage modulus of PBBMA-X is increased by 100, 260, and 340% depending on the amount of high-modulus CW added. In fact, the obtained bioplastics have a storage modulus in the range demonstrated by commercial LDPE (Figure 8 and [50,51,52]).

### 2.6. Mechanical Properties of the Bioplastics

The PBBM-X and PBBM-X/CW mechanical properties at a higher deformation level were studied through stress-strain measurements. Representative stress-strain curves are shown in Figure 8a. One can see that PBBM-X has typical behavior observed for a ductile (tough) plastic [38,53,54] with an initial linear portion, yield point, strain softening, and cold drawing with significant elongation at nearly constant draw stress. We note that the value of the draw stress is quite low, just 25% of the stress-at-yield, which is connected to the low level of entanglements in the polymer material [55,56,57]. As CW is added to PBBM-X, considerable changes in yield stress and elongation-at-yield are found. In addition, the cold drawing was not observed for the samples containing 30 and 40% of the wax. These materials are broken in the strain-softening region of the stress-strain curve.

From the stress-strain data, Young’s modulus, yield stress, and elongation-at-yield are calculated and presented in Figure 8b–d. It was found that the elastic moduli increased considerably with CW loading in comparison to the ones observed for pure PBBM-X. The modulus increases from 30 MPa to 40, 130, and 230 MPa for the bioplastics containing 15, 30, and 40% of the carnauba wax, respectively. Therefore, the modulus of the PBBM-X bioplastics with the highest CW content is comparable to the typical values of the modulus reported for LDPE (200–400 MPa) [54]. The yield stress decreases when 15% carnauba wax is added to PBBM-X. However, when more CW is added, the stress for the blends is higher than that of PBBM-X and reaches 1.6–1.7 MPa for the bioplastics with 30 and 40% of the wax, respectively. The elongation at yield decreases with the CW addition, from 17 to 3%. In general, the values of the yield stress and strain of the bioplastics are significantly lower than the typical values reported for LDPE (stress = 8–10 MPa and strain = ~20%) [54]. We associate the obtained results on the mechanical performance of the bioplastics with a low level of entanglement in the system. The materials’ performance is expected to be significantly improved with chemical cross-linking.

### 2.7. Moisture Barrier Properties

In addition to structural engineering employment, polyethylene is widely employed in packaging applications [58,59]. Specifically, LDPE is often used to coat paper as a moisture barrier [60,61]. To this end, we compared the water barrier properties of natural kraft paper covered with LDPE and the paper impregnated with CW and covered with PBBM-X/CW (40%). The measurements were conducted in a “jungle room” at 38 °C and 90% relative humidity. The water vapor transmission rate (WVTR), measured in g * m^−2^ * day^−1^, was 2100 for untreated paper. The thickness of the polyethylene coating on the kraft paper was ~35 µm. The thickness of the PBBM-X/CW film coating was 50 µm. However, significant material penetration was observed during annealing when the bioplastic film was deposited. Therefore, we impregnated the paper with CW from melt prior to the deposition of the bioplastic film. It was found that about 17.7 g/m^2^ of CW was incorporated into the paper, translated into an effective CW thickness of ~18 µm (since the density of the wax is about 1 g/cm^3^ [62]). Thus, we used 68 µm as the PBBM-X/CW thickness in our calculations. It is necessary to point out that the paper impregnated with CW had a WVTR of ~300 g * m^−2^ * day^−1^.

We determined the same values of WVTR (35 g * m^−2^ * day^−1^) for paper covered with LDPE and PBBM-X/CW. Therefore, water vapor permeability, WVP (g·100 μm·m^−2^·day^−1^·mbar^−1^) is equal to 0.21 and 0.4 for paper covered with LDPE and PBBM-X/CW, respectively. Our results demonstrate that the WVP of the bioplastic is just two times lower than that of LDPE. We also converted WVP to g * mil * m^−2^ * day^−1^ * kPa^−1^ units to directly compare our results with WVP data for various synthetic and bioderived polymers reported in a recent review by Wu et al [63] and listed in those specific units. The values are 8.2 and 15.9 g * mil * m^−2^ * day^−1^ * kPa^−1^ for the paper covered with LDPE and PBBM-X/CW, respectively. From comparing our data to that for other polymers, we found that the PBBM-X/CW combination shows the lowest WVP among biopolymers reported, with values of WVTR comparable to polyethylene terephthalate and lower than a number of synthetic materials, including nylon and polystyrene. According to Wu et al. [63], our bioplastic has “high-to-medium” water barrier performance, while most of the biodegradable polymers are classified as “low” or “poor”.

## 3. Experimental

### 3.1. Materials

Babassu oil (Dr. Adorable Inc., Chicago, IL, USA), carnauba wax (H&B Oils Center Co., Westchester, IL, USA), beechwood hemicellulose xylan with an average molecular weight of between 8000 and 10,000 g/mol provided by the supplier (RCMDT GmbH, Waghäuse, Germany), and N-(hydroxyethyl)acrylamide (TCI America) were used as received. Azobisisobutyronitrile (AIBN; Sigma-Aldrich, St. Louis, MO, USA) was purified by recrystallization from methanol. Toluene (Sigma-Aldrich, St. Louis, MO, USA) was distilled before use. Other solvents and chemicals, all of analytical grade or better, were used as received. Deionized water was used for purification purposes (MilliQ, 18 MΩ). LDPE film was obtained from Hudson Exchange, Hudson, OH. For moisture barrier experiments, 20# kraft paper was used.

### 3.2. Babassu Oil-Based Acrylic Monomer Synthesis

The synthesis of plant oil-based monomers (POBMs) is described elsewhere [15,16]. More synthetic details on the synthesis of babassu oil-based acrylic monomer (BBM) are provided in Supporting Information, Supplementary material: Sections S1 and S2.

### 3.3. Babassu Oil-Based Acrylic Monomer Characterization

To confirm the BBM chemical structure, ^1^H NMR spectra were recorded on an AVANCE III HDTM 400 high-performance digital NMR spectrometer (Bruker, Billerica, MA, USA) using CDCl_3_ as a solvent. The ESI high-resolution mass spectrometry of the BBM was obtained using a Bruker Daltonics BioTOF mass spectrometer. Iodine value (to determine the degree of BBM unsaturation), aqueous solubility, and refractive index were determined as described elsewhere [16,32].

### 3.4. Free Radical Polymerization of Babassu Oil-Based Acrylic Monomer

The average molecular weight was determined by gel permeation chromatography (GPC) using a Waters Corporation modular chromatograph consisting of a Waters 1515 HPLC pump, a Waters 2410 refractive index detector, and a set of two 10 µm PL-gel mixed-B columns; the column temperature was set at 40 °C. Tetrahydrofuran (THF) was used as the carrier solvent. The glass-transition temperature (*Tg*) of the babassu oil and BBM homopolymer was determined via differential scanning calorimetry (DSC) using TA Instruments Q2000 and Q5000 calorimeters. The samples were subjected to a heat–cool–heat process with heating and cooling rates of 10 –20 °C/min. The Tg was typically determined at the midpoint of the inflection region.

### 3.5. Maleinization of Xylan

To functionalize xylan with reactive functional groups of maleic anhydrides, an esterification procedure was adopted with some modifications [33]. To vary the degree of maleinization (DM), 1 and 2.5 wt% of xylan were dissolved in DMSO under vigorous stirring at 80 °C. Maleic anhydride was added in a molar ratio of 4:1 (based on the maleic anhydride/anhydroxylose fragment of xylan), while 0.15 wt% of KOH was used as a catalyst. The reaction was carried out for 80 min at 80 °C under continuous nitrogen flow and mechanical stirring. A threefold excess of methanol was used to precipitate maleinized xylan. To remove unreacted maleic anhydride and KOH, modified xylan was dissolved in water and precipitated using methanol. Purification was performed three times. Modified xylan was dried in an oven to a constant weight. The chemical structure of xylan and modified xylan was confirmed by ^1^H NMR spectroscopy (JEOL ECA 400 MHz NMR Spectrometer) using DMSO-d6 as a solvent.

### 3.6. Grafted Copolymerization of Maleinized Xylan and BBM

A two-step procedure was developed to polymerize BBM in the presence of maleinized xylan. First, a free radical initiator, AIBN (1.5 wt%), was dissolved in BBM. After the dissolution, maleinized xylan was gradually added to the initiator-in-monomer solution at 1:7 *w/w* and homogenized at 25,000 rpm using a mechanical mixer. Mixed material was transferred to a 3-neck round bottom flask and vigorously stirred for 8 h at 80 °C to polymerize under argon flow. To eliminate unreacted xylan and BBM, a two-step purification procedure was applied. First, the reaction mixture was precipitated using methanol to remove unreacted BBM. Afterward, toluene was used to dissolve the BBM-free reaction mixture to remove precipitated, unreacted xylan via dispersion filtration through Whatman filter paper.

### 3.7. Characterization of Xylan-g-BBM Copolymers and Copolymer/Carnauba Wax Mixtures

The glass transition temperatures of the Xylan-g-BBM copolymers and copolymer/carnauba wax mixtures were determined via DSC, as described above, using a TA Instruments Q2000 calorimeter.

Free-standing films for thermomechanical characterization were prepared using a drawdown of polymer solutions on a PTFE substrate. After casting, the films were placed in the oven at 80 °C for 1 h. Rectangular samples (5 mm in width, 15 mm in length, and 0.8–0.12 mm in thickness) were tested using dynamic mechanical analysis (DMA). The TA Instruments Q800 operated in tensile mode with a heating rate of 5 °C/min, and an oscillation frequency of 1 Hz. Stress-strain curves were recorded for the rectangular samples (5 mm in width, 15 mm in length, and 0.8–0.12 mm in thickness)at the strain rate of 5 mm/min using Instron 5542. 

### 3.8. Moisture Barrier Measurements

For moisture barrier evaluation, we used neat kraft paper, covered with LDPE film, and CW-impregnated paper covered with coatings made of PBBM-X/CW blends. CW was placed in a dish and fully melted for the paper impregnation. Following that, paper samples were dipped into molten CW. The excess CW was removed from paper surfaces so that CW was presented only within the bulk of the paper membrane. To coat the paper with PBBM-X/CW, the blend solution in toluene was deposited on a PTFE sheet with a drawdown bar. Following solvent evaporation, the coating was placed in the oven, heated up to 120 °C, and cooled down to RT (slowly in the oven), allowing film formation and crystallization to occur. Formed coatings from PBBM-X/CW blends were used to obtain free-standing films, which were deposited onto impregnated paper membranes using a silicon roller at 40–60 °C to allow film softening and ensure coated paper integrity. All the obtained samples were shaped to fit in permeability cups, while their thickness was measured with a micrometer. Permeability cups were filled with drierite to create 0% RH within the cup and a concentration gradient between the outer and inner sides of the assembly. To evaluate the water vapor transmission rate (WVTR), permeability cups were placed into a humidification chamber in an environment of 38 °C and 90% RH for 7 days (according to ASTM E96). The difference in the cup mass prior to and after the experiment was used to determine the amount of penetrated water and calculate WVTR.

## 4. Conclusions

A tough bioplastic made of a babassu oil-based acrylic polymer, hemicellulose xylan grafted with BBM chains, and carnauba wax was obtained. In order to fabricate the material, we synthesized an original acrylic monomer from babassu oil and demonstrated that a PBBM homopolymer could be obtained from BBM via radical polymerization. Xylan macromolecules decorated with reactive double bonds were also made by the reaction of xylan hydroxylic groups with maleic anhydride. Polymerization of BBBM in the presence of X-MA yielded a bioplastic mostly constituted from highly branched PBBM/X-MA macromolecules. The incorporation of X-MA into PBBM resulted in changes in the temperature transitions of the PBBM, where transitions of the side chains of BBM were shifted to higher temperatures, while Tg of the main chain decreased by about 10 °C. We found that incorporating rigid xylan chains into PBBM increased the RT storage modulus of the bioplastic by ~30%.

To increase the modulus further and approach the modulus of LDPE, CW was added to PBBM-X. We determined that PBBM-X is compatible with the wax, as evidenced by the alternation of the thermal transitions of the materials and the co-crystallization of BBM with CW. In fact, the storage modulus of the PBBM-X/CW blend, which contains 40% of the wax, is close to that of commercial LDPE. From stress-strain measurements, we established that PBBM-X and PBBM-X/CW bioplastics exhibit typical behavior for ductile (tough) plastic. It was determined that PBBM-X/(40%)CW has an elastic modulus comparable to the modulus of LDPE. However, the bioplastic’s yield strength and elongation-at-yield are considerably lower than those of polyethylene.

We evaluated the moisture barrier properties of the PBBM-X/(40%)CW material and found that the WVP of the bioplastic is approaching that of LDPE. Our bioderived material demonstrates a WVP that is comparable to polyethylene terephthalate and lower than the WVP of nylon and polystyrene. In general, we envision that the biobased polymer composition developed here has the potential to replace LDPE in a number of non-engineering packaging applications.

## Data Availability

The data that support the reported results are presented in the manuscript and Supporting Information using text, figures, and tables.

## Supplementary Materials

The following supporting information can be downloaded at: https://www.mdpi.com/article/10.3390/ijms24076103/s1. References [64,65,66] are cited in the supplementary materials.

## References

[1] Siracusa V., Blanco I. (2020). Bio-Polyethylene (Bio-PE), Bio-Polypropylene (Bio-PP) and Bio-Poly(ethylene terephthalate) (Bio-PET): Recent Developments in Bio-Based Polymers Analogous to Petroleum-Derived Ones for Packaging and Engineering Applications. Polymers.

[2] Nguyen H.T.H., Qi P., Rostagno M., Feteha A., Miller S.A. (2018). The quest for high glass transition temperature bioplastics. J. Mater. Chem. A.

[3] Plastics—The Facts 2020; Plastics Europe: Brussels, B. https://plasticseurope.org/knowledge-hub/plastics-the-facts-2020/.

[4] Lemonick S. (2018). Chemical solutions for a chemical problem. Chem. Eng. News.

[5] Shen L., Haufe J., Patel M.K. (2009). Product Overview and Market Projection of Emerging Biobased Plastics.

[6] Ebnesajjad S. (2012). Handbook of Biopolymers and Biodegradable Plastics: Properties, Processing and Applications.

[7] Rosseto M., Krein D.D., Balbé N.P., Dettmer A. (2019). Starch–gelatin film as an alternative to the use of plastics in agriculture: A review. J. Sci. Food Agric..

[8] Kimura Y. (2009). Molecular, Structural, and Material Design of Bio-Based Polymers. Polym. J..

[9] Mecking S. (2020). Polyethylene-like materials from plant oils. Philos. Trans. R. Soc. A Math. Phys. Eng. Sci..

[10] Van Beilen J.B., Poirier Y. (2012). Plants as factories for bioplastics and other novel biomaterials. Plant Biotechnology and Agriculture.

[11] Nakajima H., Kimura Y., Kimura Y. (2013). General introduction: Overview of the current development of biobased polymers. Bio-Based Polymers.

[12] Papageorgiou G. (2018). Thinking Green: Sustainable Polymers from Renewable Resources. Polymers.

[13] Caillol S. (2020). Special Issue “Natural Polymers and Biopolymers II”. Molecules.

[14] Sharmin E., Zafar F., Akram D., Alam M., Ahmad S. (2015). Recent advances in vegetable oils based environment friendly coatings: A review. Ind. Crops Prod..

[15] Tarnavchyk I., Popadyuk A., Popadyuk N., Voronov A. (2015). Synthesis and Free Radical Copolymerization of a Vinyl Monomer from Soybean Oil. ACS Sustain. Chem. Eng..

[16] Demchuk Z., Shevchuk O., Tarnavchyk I., Kirianchuk V., Kohut A., Voronov S., Voronov A. (2016). Free Radical Polymerization Behavior of the Vinyl Monomers from Plant Oil Triglycerides. ACS Sustain. Chem. Eng..

[17] Demchuk Z., Kohut A., Voronov S., Voronov A. (2018). Versatile platform for controlling properties of plant oil-based latex polymer networks. ACS Sustain. Chem. Eng..

[18] Demchuk Z., Li W.S.J., Eshete H., Caillol S., Voronov A. (2019). Synergistic Effects of Cardanol- and High Oleic Soybean Oil Vinyl Monomers in Miniemulsion Polymers. ACS Sustain. Chem. Eng..

[19] Simões A., Ramos L., Freitas L., Santos J.C., Zanin G.M., De Castro H.F. (2015). Performance of an enzymatic packed bed reactor running on babassu oil to yield fatty ethyl esters (FAEE) in a solvent-free system. Biofuel Res. J..

[20] Bauer L.C., Santos L.S., Sampaio K.A., Ferrão S.P.B., da Costa Ilhéu Fontan R., Minim L.A., Veloso C.M., Bonomo R.C.F. (2020). Physicochemical and thermal characterization of babassu oils (*Orbignya phalerata* Mart.) obtained by different extraction methods. Food Res. Int..

[21] Greenberg S.A., Alfrey T. (1954). Side Chain Crystallization of n-Alkyl Polymethacrylates and Polyacrylates^1^. J. Am. Chem. Soc..

[22] Wiley R.H., Brauer G.M. (1948). Refractometric determination of second-order transition temperatures in polymers. III. Acrylates and methacrylates. J. Polym. Sci..

[23] Collins T., Gerday C., Feller G. (2005). Xylanases, xylanase families and extremophilic xylanases. FEMS Microbiol. Rev..

[24] Scheller H.V., Ulvskov P. (2010). Hemicelluloses. Annu. Rev. Plant Biol..

[25] Beg Q.K., Kapoor M., Mahajan L., Hoondal G.S. (2001). Microbial xylanases and their industrial applications: A review. Appl. Microbiol. Biotechnol..

[26] Polizeli M., Rizzatti A.C.S., Monti R., Terenzi H.F., Jorge J.A., Amorim D.S. (2005). Xylanases from fungi: Properties and industrial applications. Appl. Microbiol. Biotechnol..

[27] Milanovic J., Manojlovic V., Levic S., Rajic N., Nedovic V., Bugarski B. (2010). Microencapsulation of Flavors in Carnauba Wax. Sensors.

[28] De Freitas C.A.S., de Sousa P.H.M., Soares D.J., da Silva J.Y.G., Benjamin S.R., Guedes M.I.F. (2019). Carnauba wax uses in food—A review. Food Chem..

[29] Lim J., Hwang H.-S., Lee S. (2017). Oil-structuring characterization of natural waxes in canola oil oleogels: Rheological, thermal, and oxidative properties. Appl. Biol. Chem..

[30] Despond S., Espuche E., Cartier N., Domard A. (2005). Barrier properties of paper–chitosan and paper–chitosan–carnauba wax films. J. Appl. Polym. Sci..

[31] Dos Santos F.K.G., Silva K.N.D.O., Xavier T.D.N., Leite R.H.D.L., Aroucha E.M.M. (2017). Effect of the Addition of Carnauba Wax on Physicochemical Properties of Chitosan Films. Mater. Res..

[32] Kohut A., Voronov S., Demchuk Z., Kirianchuk V., Kingsley K., Shevchuk O., Caillol S., Voronov A. (2020). Non-Conventional Features of Plant Oil-Based Acrylic Monomers in Emulsion Polymerization. Molecules.

[33] Peng X.-W., Ren J.-L., Sun R.-C. (2010). Homogeneous Esterification of Xylan-Rich Hemicelluloses with Maleic Anhydride in Ionic Liquid. Biomacromolecules.

[34] Iyer K.S., Luzinov I. (2004). Effect of Macromolecular Anchoring Layer Thickness and Molecular Weight on Polymer Grafting. Macromolecules.

[35] Hiemenz P.C., Lodge T. (2007). Polymer Chemistry.

[36] Nassu R.T., Guaraldo Gonçalves L.A. (1999). Determination of melting point of vegetable oils and fats by differential scanning calorimetry (DSC) technique. Grasas Aceites.

[37] Sperling L.H. (2006). Introduction to Physical Polymer Science.

[38] Fried J.R. (2014). Polymer Science and Technology.

[39] Wei L., Caliskan T.D., Tu S., Choudhury C.K., Kuksenok O., Luzinov I. (2020). Highly Oil-Repellent Thermoplastic Boundaries via Surface Delivery of CF3 Groups by Molecular Bottlebrush Additives. ACS Appl. Mater. Interfaces.

[40] Bongiovanni R., Malucelli G., Lombardi V., Priola A., Siracusa V., Tonelli C., Di Meo A. (2001). Surface Properties of Methacrylic Copolymers Containing a Perfluoropolyether Structure. Polymer.

[41] Bongiovanni R., Nelson A., Vitale A., Bernardi E. (2012). Ultra-thin Films Based on Random Copolymers Containing Perfluoropolyether Side Chains. Thin Solid Films.

[42] Krupers M., Slangen P.-J., Möller M. (1998). Synthesis and Properties of Polymers Based on Oligo(hexafluoropropene oxide) Containing Methacrylates and Copolymers with Methyl Methacrylate. Macromolecules.

[43] Pelras T., Mahon C.S., Müllner M. (2018). Synthesis and Applications of Compartmentalised Molecular Polymer Brushes. Angew. Chem. Int. Ed..

[44] Tu S.D., Choudhury C.K., Luzinov I., Kuksenok O. (2019). Recent advances towards applications of molecular bottlebrushes and their conjugates. Curr. Opin. Solid State Mat. Sci..

[45] Daniel W.F.M., Burdyńska J., Vatankhah-Varnoosfaderani M., Matyjaszewski K., Paturej J., Rubinstein M., Dobrynin A.V., Sheiko S.S. (2016). Solvent-free, supersoft and superelastic bottlebrush melts and networks. Nat. Mater..

[46] Kunamaneni S., Buzza D.M.A., Read D.J., Parker D., Kenwright A.M., Feast W.J., Larsen A.L. (2006). Entanglement Transition of Randomly Branched Polymers in the Hyperbranched Class. Macromolecules.

[47] Lusignan C.P., Mourey T.H., Wilson J.C., Colby R.H. (1995). Viscoelasticity of randomly branched polymers in the critical percolation class. Phys. Rev. E.

[48] Colby R.H., Gillmor J.R., Rubinstein M. (1993). Dynamics of near-critical polymer gels. Phys. Rev. E.

[49] Lusignan C.P., Mourey T.H., Wilson J.C., Colby R.H. (1999). Viscoelasticity of randomly branched polymers in the vulcanization class. Phys. Rev. E.

[50] Mohagheghian I., McShane G.J., Stronge W.J. (2015). Impact perforation of monolithic polyethylene plates: Projectile nose shape dependence. Int. J. Impact Eng..

[51] Salakhov I.I., Shaidullin N.M., Chalykh A.E., Matsko M.A., Shapagin A.V., Batyrshin A.Z., Shandryuk G.A., Nifant’Ev I.E. (2021). Low-Temperature Mechanical Properties of High-Density and Low-Density Polyethylene and Their Blends. Polymers.

[52] Liang J.-Z. (2010). Predictions of Storage Modulus of Glass Bead-Filled Low-Density-Polyethylene Composites. Mater. Sci. Appl..

[53] Van Krevelen D.W. (2000). Properties of Polymers: Their Correlation with Chemical Structure, Their Numerical Estimation and Prediction from Additive Group Contributions.

[54] Ehrenstein G.W. (2001). Polymeric Materials: Structure, Properties, Applications.

[55] Men Y., Rieger J., Strobl G. (2003). Role of the Entangled Amorphous Network in Tensile Deformation of Semicrystalline Polymers. Phys. Rev. Lett..

[56] Termonia Y., Smith P. (1987). Kinetic model for tensile deformation of polymers. Macromolecules.

[57] Qian R. (1997). The concept of cohesional entanglement. Macromol. Symp..

[58] Marsh K., Bugusu B. (2007). Food Packaging: Roles, Materials, and Environmental Issues. J. Food Sci..

[59] Jagadish R.S., Raj B., Asha M.R. (2009). Blending of low-density polyethylene with vanillin for improved barrier and aroma-releasing properties in food packaging. J. Appl. Polym. Sci..

[60] Pawelec W., Tirri T., Aubert M., Häggblom E., Lehikoinen T., Skåtar R., Pfaendner R., Wilén C.-E. (2015). Toward halogen-free flame resistant polyethylene extrusion coated paper facings. Prog. Org. Coat..

[61] Lahtinen K., Kuusipalo J. (2008). Statistical prediction model for water vapor barrier of extrusion-coated paper. Tappi J..

[62] https://www.chemsrc.com/en/cas/8015-86-9_1198975.html.

[63] Wu F., Misra M., Mohanty A.K. (2021). Challenges and new opportunities on barrier performance of biodegradable polymers for sustainable packaging. Prog. Polym. Sci..

[64] Odian G.G. (2004). Principles of Polymerization.

[65] Ravishankar K., Shelly K.M., Desingh R.P., Subramaniyam R., Narayanan A., Dhamodharan R. (2018). Green, Solid-State Synthesis of Maleated Chitosan and Ionotropic Gelation with Chitosan. ACS Sustain. Chem. Eng..

[66] Kujawa J., Rynkowska E., Fatyeyeva K., Knozowska K., Wolan A., Dzieszkowski K., Li G., Kujawski W. (2019). Preparation and Characterization of Cellulose Acetate Propionate Films Functionalized with Reactive Ionic Liquids. Polymers.

