# Supplementary material for: Tough Bioplastics from Babassu Oil-Based Acrylic Monomer, Hemicellulose Xylan, and Carnauba Wax
Source: Int J Mol Sci. 2023 Mar 23;24(7):6103. doi: 10.3390/ijms24076103 (PMC10094404; doi:10.3390/ijms24076103)
Supplement: Supplementary file 1 [file ijms-24-06103-s001.zip › ijms-2270010-supplementary.pdf]

Supporting Information

Tough Bioplastics from Babassu Oil-Based Acrylic Monomer, Hemicellulose Xylan, and Carnauba Wax

Yehor Polunin,[1] Vasylyna Kirianchuk,[1] Najah Mhesn,[2] Liying Wei,[2] Sergiy Minko,[3]

Igor Luzinov,[2*] and Andriy Voronov[1*]

[1]Department of Coatings and Polymeric Materials, North Dakota State University, Fargo, North Dakota 58105, USA
[2]Department of Materials Science and Engineering, Clemson University, Clemson, South Carolina 29634, USA
[3]Nanostructured Materials Laboratory, University of Georgia, Athens, Georgia, 30602 USA

*To whom correspondence should be addressed: luzinov@clemson.edu and andriy.voronov@ndsu.edu

S1: Synthesis and Characterization of Acrylic Monomer from Babassu Oil (BBM)

S2. Free Radical Polymerization of BBM

S3. Characterization of PBBM homopolymer

S4: NMR spectra of maleinized xylan

S1: Synthesis and Characterization of Acrylic Monomer from Babassu Oil (BBM)

The synthesis of different plant oil-based acrylic monomers (POBM) was previously reported by us. [1-3] In brief, about 115 g of N- (hydroxyethyl) acrylamide was added to 150 g of babassu oil (with acrylamide alcohol to triglyceride molar ratio of 5.9 to 1), 150 mL of tetrahydrofuran, and 0.1 g of 2,6-ditertbutyl-p-cresol in a 500 mL round-bottomed flask equipped with a mechanical stirrer. The reaction mixture was heated to 50 °C in the presence of a catalytic amount of potassium hydroxide (1.5 g), which was slowly added to the reaction mixture with continuous stirring. The reaction mixture was stirred at 50 °C for 4 h until complete transesterification. The reaction mixture was diluted with CH_2Cl_2, purified by washing with brine, treated with magnesium sulfate, and dried under a vacuum, yielding about 165 g of acrylic monomer (94–96% of the theoretical yield).

The FTIR spectrum indicates the addition of fatty acid acyl moieties to the acrylamide fragment (**Figure S1 A**). Strong NH absorption band at 3200-3400 cm^{-1}, the carbonyl (amide I) band at 1670, and the NH (amide II) band at 1540 cm^{-1} confirm the attachment of the acrylamide species to the fatty fragment. The presence of the strong bands at 1740, 1245, and 1180 cm^{-1} indicates the ester nature of the synthesized monomer.

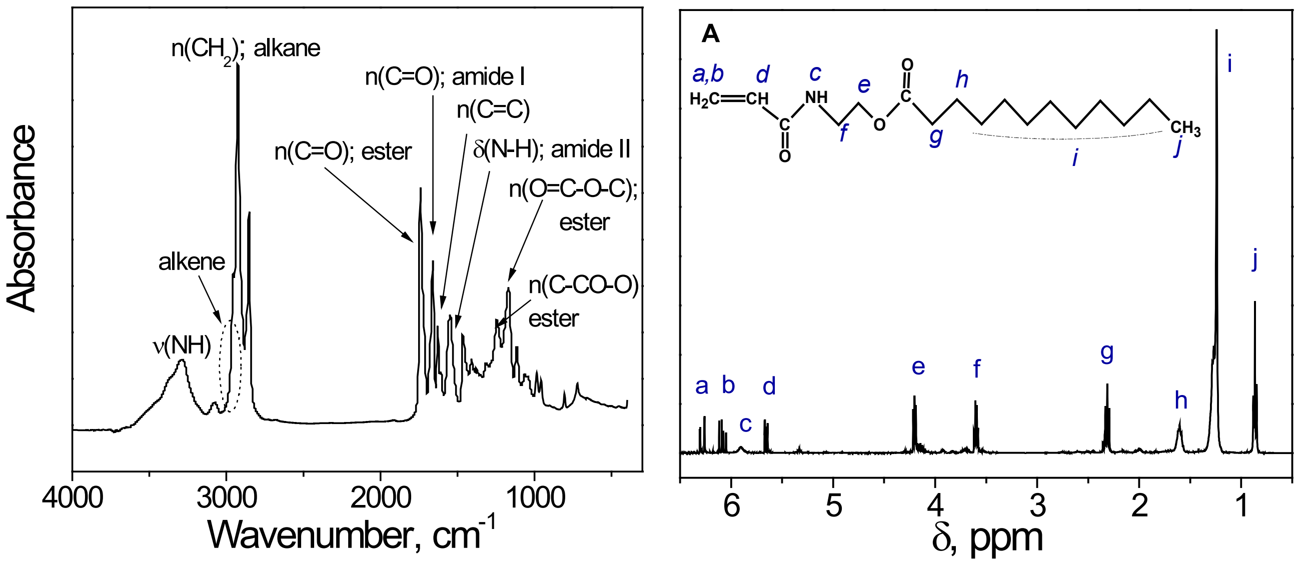

Figure S1. FT-IR (**A**) and ^{1}H NMR (**B**) spectroscopy for BBM.

Figure S1 B shows the ^{1}H-NMR spectrum of BBM, confirming the presence of the characteristic peaks of the protons of the acrylic double bond at 5.6-6.6 ppm (peeks *a*, *b*, and *d*), the peaks of the protons of the ethylene linkage between the amide and ester groups at 3.6 and 4.20 ppm (peeks *f* and *e* respectively), and signals from the fatty acid chains (0.8 to 2.8 ppm). Barely noticeable characteristic peaks at 5.3 ppm indicate the presence of unsaturated fatty acid (oleic acid) fragments in the monomer mixture. Similar spectra were previously recorded for other POBMs confirming the acryloylamide moiety's presence in their molecules. This allows for predicting similar reactivity of the double bond in the BBM acryloylamide moiety in radical polymerization. [1,2]

Characteristic ESI mass spectrum of BBM shows the highest peak at 320 m/z (**Figure S2**), corresponding to [laurate-BBM+ Na]$^+$ fraction, which correlates with the fatty acid composition of babassu oil where the amount of lauric acid fragments is around 50 % wt. This demonstrates that the majority of BBM is (acryloylamino)ethyl laurate. To determine the molecular weight of POBM, the subtraction of the mass of the sodium ion was applied. After calculations, the molecular weight of BBM

was defined as 297. Additionally, BBM molecules consist of small fractions of other fatty acid chains confirmed by mass peaks at 264, 348, 376, and 402 m/z The molecular weight of the BBM is in good agreement with the theoretical average molecular weight. Overall, the mass spectrum shows that monomers in the resulting mixture contain one acrylic double bond linked to one fatty chain, which varied between fully saturated and monounsaturated fragments of babassu oil.

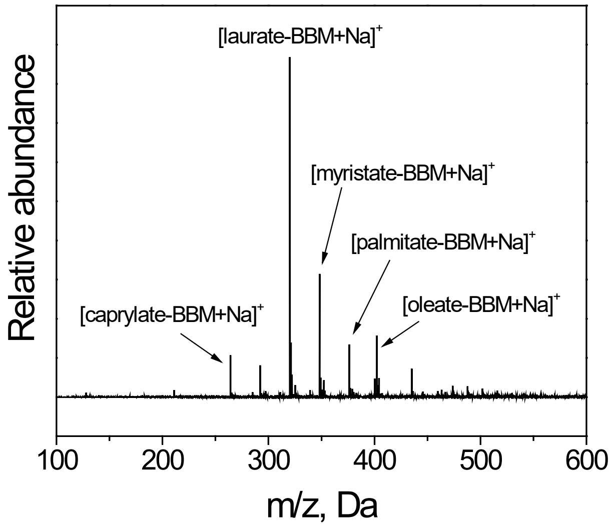

Figure S2. ESI mass spectra of BBM.

To characterize BBM's degree of unsaturation, the iodine value (IV) was determined (amount of iodine in g that reacts with fatty acid chain double bonds at specified conditions) and compared to the IV of other POBMs. The obtained values (IV_{BBM} = 56 g/g, $IV_{babassu\ oil}$ = 12 g/g) confirm the chemical composition of BBM containing predominantly saturated fatty acid fractions.

Using standard procedures, the density (d^{20} = 0.948 g/cm^3) and refractive index (n^{20}=1.460) of BBM were determined, as well as its aqueous solubility (10^{-3} mol/l), confirming BBM's highly hydrophobic nature.

Figure S3 shows DSC traces for babassu oil and BBM.

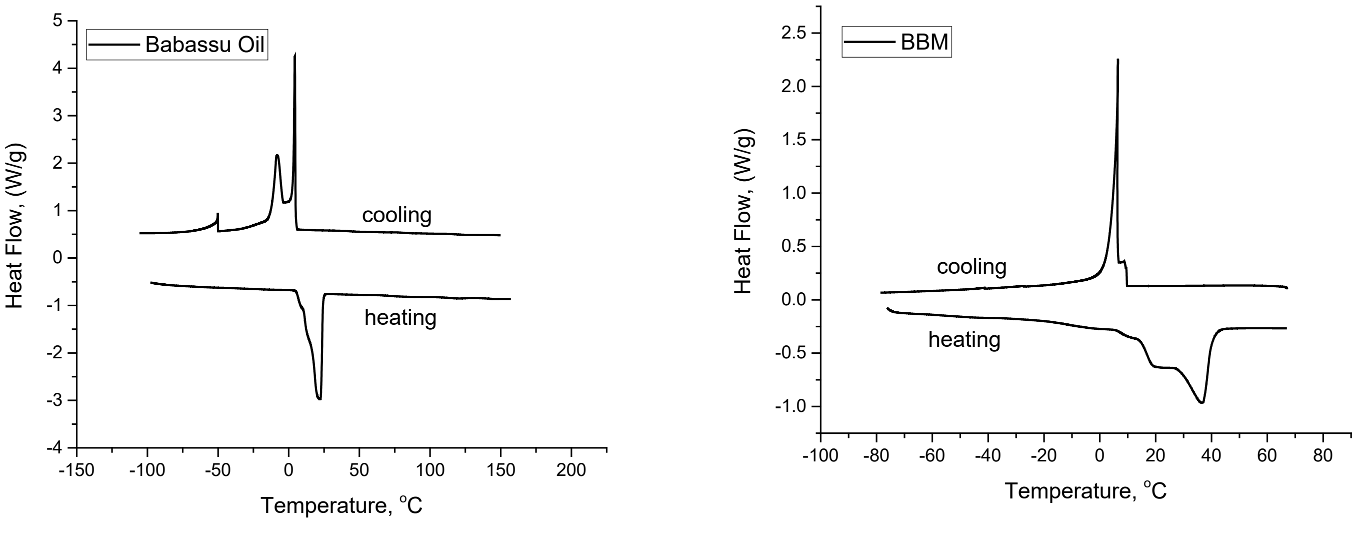

Figure S3. DSC thermograms of the babassu oil and BBM.

S2. Free Radical Polymerization of BBM

To establish the BBMs' capability for free radical reactions and investigate the effect of monomer structure on reaction rate, homopolymerization was carried out in solution. The free radical polymerization rate is described by equation (**1**), showing a first-order dependence of R_p on the monomer concentration and square root dependence on the initiator concentration.[4]

$$R_p = -\frac{d[\mathrm{M}]}{dt} = k_p\left(\frac{k_d f[\mathrm{I}]}{k_t}\right)^{1/2}[\mathrm{M}] \quad \textbf{(Eq. S1)}$$

where [M] and [I] are the monomer and initiator concentrations, respectively, f is the initiator efficiency, k_d is the rate constant of initiator decomposition, k_p is the propagation rate constant, and k_t is the rate constant for termination.

To determine the order of reaction with respect to the initiator, polymerization was conducted at a constant BBM concentration ([M] = 0.2 M) and varied AIBN initiator concentrations ([I] = 0.05–0.08 M) at 70 °C. For each initiator concentration, the polymerization rate (Rp, mol L^{-1} s^{-1}) was determined on a linear part of the conversion vs. time plot (up to a 10–15% monomer conversion). For this purpose, polymerization yield was calculated gravimetrically by precipitation of a fraction of the reaction mixture in methanol. The obtained product was centrifuged in a weighted tube at 4000 rpm for 5–10 min to ensure complete precipitation of the polymer. The supernatant was poured out, and the precipitate was dried in a vacuum until constant weight. The ln Rp was plotted against ln ([I]), and a slope of the straight line (i.e., tan α, which corresponds to the order of reaction with respect to the initiator) was calculated (**Figure S4 A**) to yield a value of 0.79. It is in line with the previous research, the BBM as a predominantly saturated monomer has the lowest order with respect to the initiator compared with data for the library of POBMs. [1,2]

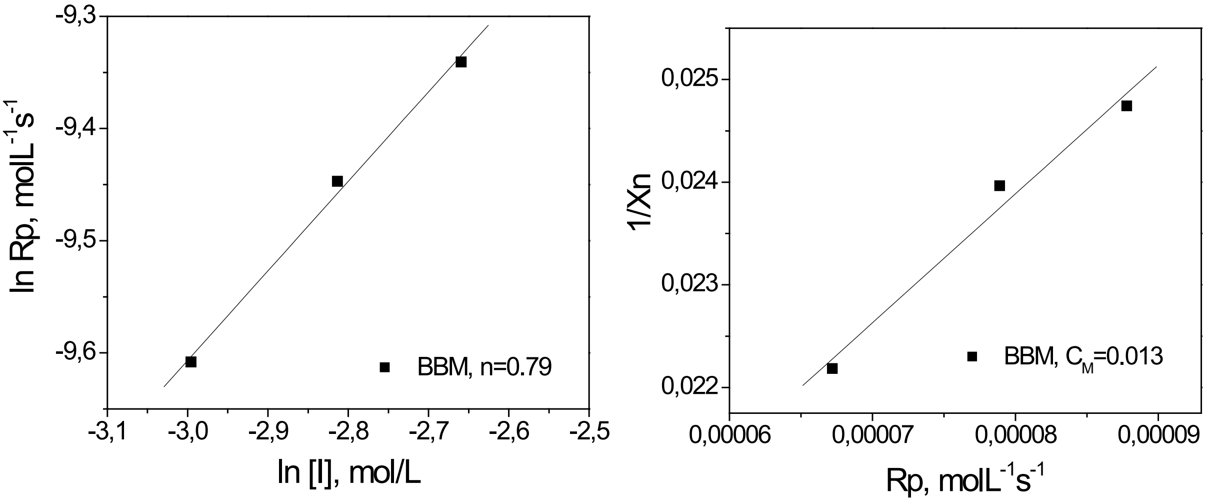

Figure S4. Polymerization rate of the BBM vs. [I] at 70°C (**A**) and chain transfer constant on BMM determined using Mayo method (**B**).

The dependence of R_p on $[\mathrm{I}]^{1/2}$ is a consequence of a bimolecular termination reaction between growing radicals (macroradicals). In the case of BBM, higher than 0.5 order of R_p on [I] is assigned to, the so-called premature termination (chain transfer reactions) of a growing macroradical by transfer of

hydrogen from allylic unsaturated fragments of oleic acid as this mechanism has been confirmed and reported previously.[1,2]

To evaluate the extent of the effect of chain transfer on polymerization, the Mayo method was employed to determine the values of chain transfer constants on BBM (C_M) in polymerization at 70 °C. In this method, the inverse value of the number average degree of polymerization is plotted against the polymerization rate, and C_M is determined by the intercept by extrapolating to zero rate (**Figure S3 B**). The obtained value of C_M is low (C_M=0.013) (**Figure S3 B**), thus indicating that chain propagation during polymerization of BBM is not significantly impacted by effective chain transfer to monomer.

S3. Characterization of PBBM homopolymer

The ^{1}H NMR spectrum of BBM homopolymer (**Figure S5**) confirms the absence of vinyl double bonds of the acrylic functional groups taking part in the formation of macromolecules from the BBM repeating units.

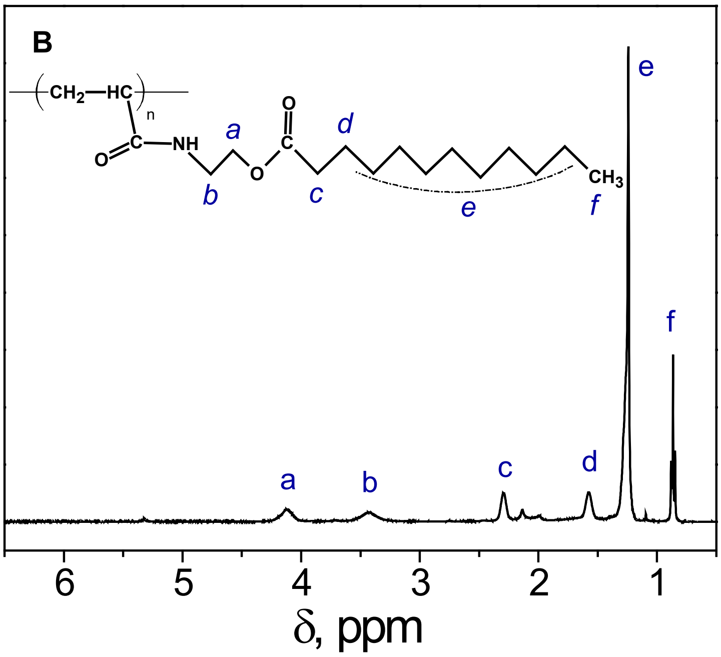

Figure S5. ^{1}H NMR spectrum of PBBM.

S4: NMR spectra of maleinized xylan

Degree of maleinization (DM) is a quantitative characteristic of modification extent, which determines the number of maleic anhydride moieties per anhydroglucose unit of hemicellulose. Using normalized, integrated intensities of signal I_1 from 1 proton of anhydroglucose unit (4.15-4.3 ppm) and I_2 from 3 protons of maleoylated moiety (5.9-6.55 ppm), DM of maleinized xylan using equation (1) [5,6]:

$$DS = \frac{I_2 * 1}{I_1 * 3} \quad (1)$$

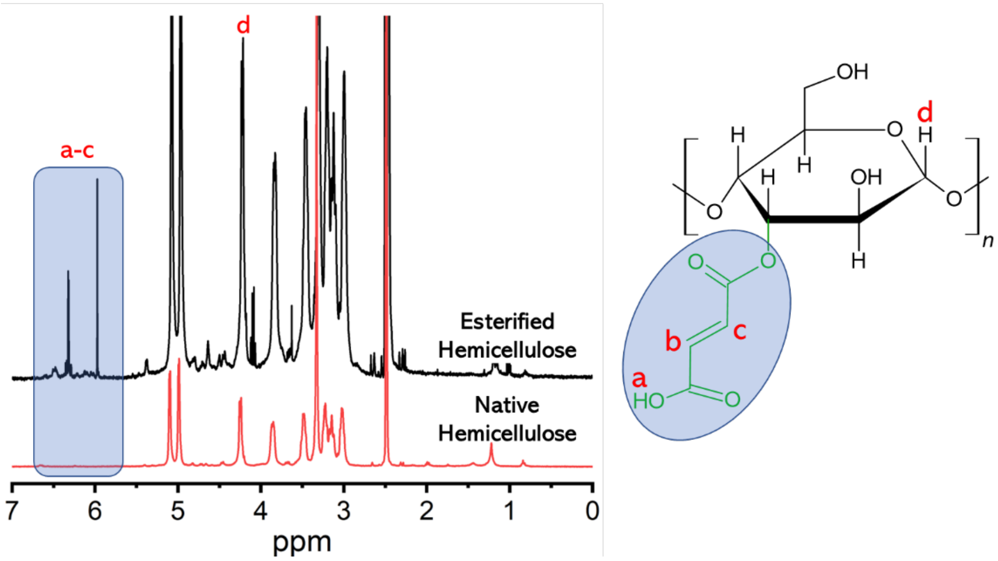

Figure S6. ^{1}H NMR spectra of xylan (red) and modified xylan (black).

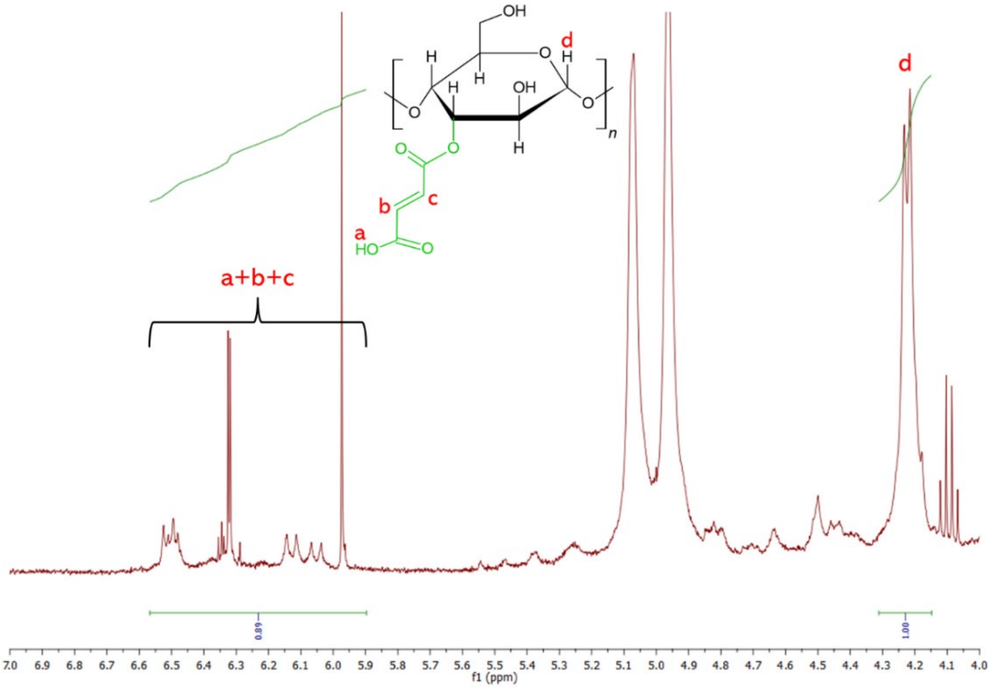

Figure S7. ^{1}H NMR spectrum of maleinized xylan (degree of maleinization 0.3).

References

1. Demchuk, Z.; Shevchuk, O.; Tarnavchyk, I.; Kirianchuk, V.; Kohut, A.; Voronov, S.; Voronov, A. Free Radical Polymerization Behavior of the Vinyl Monomers from Plant Oil Triglycerides. *ACS Sustainable Chemistry & Engineering* **2016**, *4*, 6974-6980, doi:10.1021/acssuschemeng.6b01890.
2. Tarnavchyk, I.; Popadyuk, A.; Popadyuk, N.; Voronov, A. Synthesis and Free Radical Copolymerization of a Vinyl Monomer from Soybean Oil. *ACS Sustainable Chemistry & Engineering* **2015**, *3*, 1618-1622, doi:10.1021/acssuschemeng.5b00312.
3. Demchuk, Z.; Kohut, A.; Voronov, S.; Voronov, A. Versatile platform for controlling properties of plant oil-based latex polymer networks. *ACS Sustainable Chemistry & Engineering* **2018**, *6*, 2780-2786, doi:10.1021/acssuschemeng.7b04462.
4. Odian, G.G. *Principles of Polymerization*; Wiley-Interscience: Hoboken, N.J., 2004.
5. Ravishankar, K.; Shelly, K.M.; Desingh, R.P.; Subramaniyam, R.; Narayanan, A.; Dhamodharan, R. Green, Solid-State Synthesis of Maleated Chitosan and Ionotropic Gelation with Chitosan. *ACS Sustainable Chemistry & Engineering* **2018**, *6*, 15191-15200, doi:10.1021/acssuschemeng.8b03648.
6. Kujawa, J.; Rynkowska, E.; Fatyeyeva, K.; Knozowska, K.; Wolan, A.; Dzieszkowski, K.; Li, G.; Kujawski, W. Preparation and Characterization of Cellulose Acetate Propionate Films Functionalized with Reactive Ionic Liquids. *Polymers* **2019**, *11*, 1217, doi:10.3390/polym11071217.
